# Incidence and Characteristics of ST-Elevation Myocardial Infarction in Patients With Previous Coronary Artery Bypass Grafting: A Single-Center Experience

**DOI:** 10.7759/cureus.86978

**Published:** 2025-06-29

**Authors:** Ilgizar Hart, Vivek Bhat, Sayyad Kyazimzade, Eric Taylor, Ester Masati, Phuong N Le, Hayden Braun, Christopher Nielsen, Valerian Fernandes

**Affiliations:** 1 Department of Medicine, Medical University of South Carolina, Charleston, USA; 2 Department of Internal Medicine, State University of New York Upstate Medical University, Syracuse, USA

**Keywords:** acute coronary bypass failure, acute coronary syndrome (acs) and stemi, pci to svg, post-cabg stemi, protective lima

## Abstract

Introduction: ST-elevation myocardial infarction (STEMI) in patients with prior coronary artery bypass graft (CABG) represents a complex entity, with limited characterization. We sought to analyze the clinical and angiographic characteristics of post-CABG patients presenting with STEMI at a single, large referral center in the Southeastern United States.

Methods: We reviewed the STEMI alert database at the Medical University of South Carolina (MUSC). We included all patients with STEMI, as defined by the Fourth Universal Definition of Myocardial Infarction, who presented between June 2009 and December 2022. From this, we then identified those with prior CABG and analyzed their clinical and angiographic features. We compared these characteristics with patients without prior CABG. Finally, we analyzed outcomes including in-hospital complications, length of stay (LOS), in-hospital mortality, and one-year mortality.

Results: Of 836 true STEMIs, 35 (4.2%) patients had a previous CABG. The mean (standard deviation (SD)) age was 67 (12) years, and most were male patients with multiple comorbidities. Thirty-one (88.6%) underwent percutaneous coronary intervention (PCI), of which 14 (45.2%) were due to acute graft occlusion. The most common graft involved was the saphenous vein graft (SVG), seen in 13 (41.9%), while multiple-vessel PCI was the most common in those with native vessel disease, seen in six (19.4%). Most patients achieved thrombolysis in myocardial infarction (TIMI) grade 3 revascularization. In-hospital mortality occurred in two (5.7%) patients, and the mean length of stay was five days. Compared to patients without prior CABG, they were older and had greater comorbidity burdens, but survival did not differ significantly.

Conclusion: Patients with prior CABG constitute the minority of patients presenting with STEMI. Post-CABG patients were typically older and had a greater comorbidity burden than those without prior CABG. Clinical outcomes after PCI were similar between the two groups, although cautious interpretation is warranted, given the small sample and observational nature of our study.

## Introduction

With advances in age and an increase in comorbidities, the complexity of patients presenting with ST-elevation myocardial infarction (STEMI) has been increasing [1]. Patients with prior coronary artery bypass graft (CABG) surgery represent one such challenging population. Immediate PCI remains the treatment of choice even for CABG patients presenting with ST-elevation myocardial infarction [2].

Patients with prior CABG represent a small proportion of STEMI patients [1,3], but their outcomes have been demonstrated to be worse than in patients without prior CABG [4-6]. Further, among patients with CABG, most acute coronary syndrome (ACS) presentations are not of STEMI [7]. Thus, there remains limited characterization of STEMI in patients with previous CABG.

The primary objective of our study is to analyze the characteristics of STEMI in patients with previous CABG, presenting at the Medical University of South Carolina (MUSC). We hypothesized that patients with prior CABG suffer from STEMI less frequently than patients without a history of coronary bypass.

## Materials and methods

The Medical University of South Carolina (MUSC) is a quaternary care university hospital in South Carolina, United States. It is the major referral center for the state and one of the largest in the Southern United States. MUSC's Chest Pain Center is a nationally accredited center for the comprehensive diagnosis and treatment of chest pain, to which patients with acute chest pain or other symptoms concerning for ACS are brought by emergency services. All patients with a STEMI alert are then examined and taken up for emergent coronary angiography.

The MUSC STEMI alert database records all patients presenting with concerns about STEMI in the Epic electronic medical record system. We conducted a retrospective observational cohort study based on the available STEMI alert database. In this retrospective observational cohort study, we examined all patients in this database, presenting from June 2009 to December 2022.

The inclusion criteria for patients were the presence of STEMI, which was identified as a cluster of four clinical syndromes using the universal definition of myocardial infarction [8]. First, symptoms of acute myocardial ischemia included the sudden onset of crushing chest pain, shortness of breath (dyspnea), sweating (diaphoresis), or cardiac arrest. Second, EKG changes indicative of STEMI include new ST-segment elevation of at least 0.1 mV in two contiguous leads, except in leads V2 and V3, where the elevation should be 0.2 mV in men and 0.15 mV in women, or the presence of a new left bundle branch block (LBBB), or changes that meet Sgarbossa's criteria in patients who already have pre-existing LBBB. Third, cardiac biomarkers were considered, with troponin I measurements being relevant from 2009 to 2020 and high-sensitivity troponin from 2021 to 2022, with a rise and fall pattern. The final gold standard is coronary angiography, which identifies acute thrombotic occlusion of the culprit's vessel, either a coronary artery or a bypass graft, including vein grafts or internal mammary artery bypass grafts. We included patients who satisfied those criteria in our analysis. We further stratified patients into two cohorts: those with prior CABG and those who did not have prior CABG.

Data collected included demographic details, comorbidities, clinical manifestations, angiographic details, and clinical outcomes, which included hospital course, length of stay (LOS), and 30-day mortality. Angiographic images were characterized by identifying the culprit lesion (thrombotic occlusion) of the native artery or graft, and the thrombolysis in myocardial infarction (TIMI) flow score was used to evaluate restoration of the flow. Successful PCI was defined as restoration of TIMI grade 3 flow with <30% residual stenosis. Evaluations were made at the time of angiography by the operator performing procedures and confirmed during review of the chart. Continuous variables are presented as means (standard deviation (SD)) or medians (interquartile range (IQR)), while categorical variables are presented as percentages. When specific data points were unavailable (noted as u/n in the Appendices), patients were excluded from relevant sub-analyses.

We then compared characteristics of STEMI patients with prior CABG to those without prior CABG, using appropriate inferential analyses: unpaired t-test for continuous variables, Chi-square test for categorical variables, and Fisher's exact test for categorical variables with small samples. We used Kaplan-Meier survival analysis to estimate the one-year survival rate in each group. All p-values were two-sided, with p<0.05 considered statistically significant. All analyses were carried out using GraphPad (Dotmatics, Boston, MA).

This study was approved by the MUSC Institutional Review Board (IRB) (reference: Pro00094039).

## Results

The MUSC STEMI activation database had 1,114 activations within the study period. A total of 1,080 were taken to the cardiac catheterization laboratory, 836 (75.04%) of which were confirmed angiographic STEMI. Thirty-five (4.19%) of these patients had a prior CABG.

Characteristics of STEMI in patients with previous CABG

The mean (SD) age was 67 (12) years. The vast majority were male patients (N=27, 77.1%) and Caucasian (N=32, 91.4%), with hypertension (N=31, 88.6%), hyperlipidemia (N=29, 82.9%), and smoking (N=25, 71.4%) seen in most of them. The mean (SD) body mass index (BMI) was 28.3 (4) kg/m^2^. The mean (SD) hemoglobin was 12.4 (2.2) gm/dL. Three (8.6%) had a history of at least one PCI before their CABG, while 11 (31.4%) had a history of PCI after CABG, but before their current presentation. Four (11.4%) had prior heart failure, three (8.6%) had atrial fibrillation, 10 (28.5%) had peripheral artery disease, three (8.6%) had prior stroke, seven (20%) had prior cancer, and three (8.6%) had liver cirrhosis. More than half (N=18, 51.4%) were on statins, six (17.1%) had a history of statin intolerance, and medication data were unavailable for nine patients.

The median (IQR) time to STEMI after CABG was 11 (13) years, ranging from one day post-CABG to 25 years. The most common site of STEMI was the inferior wall in 21 (60%) patients. Ten (28.6%) patients had anterior or anterolateral wall STEMI, whereas posterior wall STEMI was seen in four (11.4%) patients. Four (11.4%) patients presented with cardiac arrest and required resuscitation before undergoing left heart catheterization (Appendices). Thirty-one (88.6%) patients underwent PCI. Fourteen (45.2%) patients required PCI of bypass grafts, while 17 (54.8%) required PCI of native vessels.

**Figure 1:**
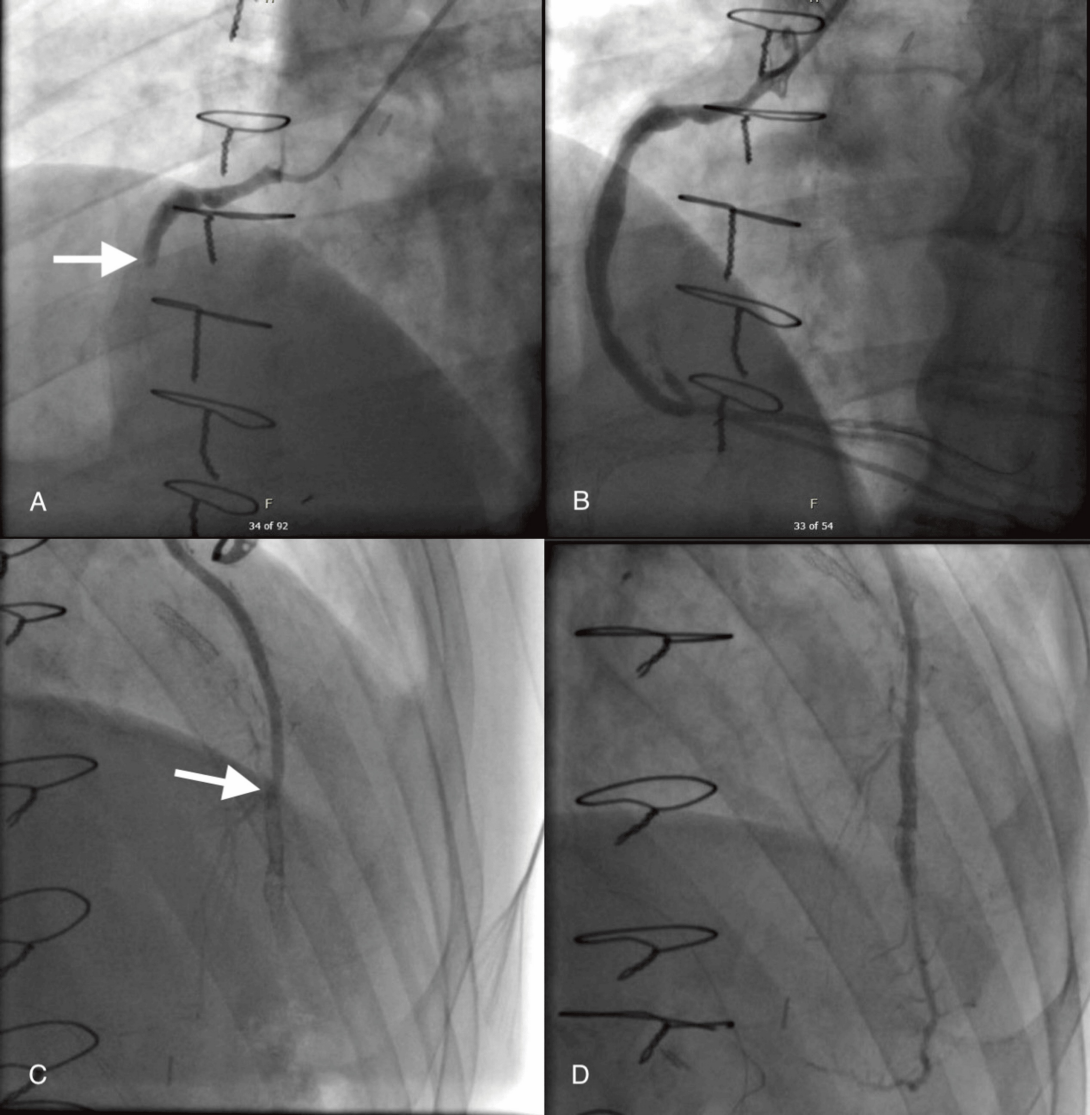
Angiographic images of graft-vessel PCI presenting with STEMI Thrombotic occlusion of the venous graft to the RCA (white arrow points to the occlusion site) (A). Successful PCI of the occluded venous graft to the RCA (B). Occluded LIMA due to prior stent with strut fracture at LAD anastomosis (white arrow points at the occlusion site) (C). Successful PCI of the LIMA occlusion (D). Images provided to illustrate the culprit vessel in STEMI with prior CABG CABG: coronary artery bypass graft, LAD: left anterior descending, LIMA: left internal mammary artery, PCI: percutaneous coronary intervention, RCA: right coronary artery, STEMI: ST-elevation myocardial infarction

Of those who underwent bypass graft PCI, the most common type of graft involved was saphenous vein graft (SVG) to the right coronary artery (RCA), seen in four (12.9%) patients. One patient had a left internal mammary artery (LIMA) graft occlusion due to a stent fracture from a previous PCI of the left anterior descending (LAD) artery, distal to the anastomosis. Figures 1A-1D highlight these cases. Of those who underwent native vessel PCI, multivessel PCI (N=6, 19.4%) and RCA (N=5, 16.1%) were the most common (Table 1).

**Table 1:** Culprit vessel in post-CABG patients with STEMI Expressed as a percentage of those undergoing PCI CABG: coronary artery bypass graft, D1: first diagonal artery, LAD: left anterior descending artery, LIMA: left internal mammary artery, OM: obtuse marginal artery, PCI: percutaneous coronary intervention, PDA: posterior descending artery, RCA: right coronary artery, STEMI: ST-elevation myocardial infarction, SVG: saphenous vein graft

Graft PCI	Number (%)
SVG-RCA	4 (12.9%)
SVG-PDA	3 (9.7%)
SVG-OM	3 (9.7%)
SVG-D1	2 (6.5%)
SVG-Ramus	1 (3.2%)
LIMA-LAD	1 (3.2%)
Native vessel PCI	Number (%)
Multiple vessels	6 (19.3%)
RCA	5 (16.1%)
LAD	4 (12.9%)
LCX	1 (3.2%)
D1	1 (3.2%)

Among 31 CABG patients who underwent PCI, successful revascularization was achieved in 29 (93.5%) patients. One patient had TIMI grade 1 flow (PCI to SVG-PDA), while the other had TIMI grade 2 flow (PCI to SVG-OM) (Appendices). In one patient, the lesion was not amenable to being crossed with a wire either through native RCA or SVG, so PCI was not performed. Another patient became hemodynamically unstable during a PCI attempt to RCA, requiring urgent mechanical circulatory support (MCS), PCI was aborted, and the RCA lesion was later found to be chronic in nature. Among these patients, all but one were treated with dual antiplatelet therapy (DAPT) with aspirin and clopidogrel, while the last patient was treated with clopidogrel and apixaban.

During hospitalization, five (14.3%) patients developed cardiogenic shock, six (17.1%) required temporary MCS with intra-aortic balloon pump (IABP) placement, five (14.3%) developed acute heart failure exacerbation, four (11.4%) developed an acute kidney injury (AKI), three (8.6%) developed an access site hematoma, and one (2.9%) had severe access site bleeding. Further, four (11.4%) patients developed pneumonia, two (5.7%) developed urinary tract infection, and three (8.6%) developed transitory encephalopathy. Two patients died during hospitalization. One initially presented with cardiac arrest (pulseless electrical activity) and was successfully resuscitated. He was taken to the catheterization laboratory, but during coronary angiography, no targets were identified for intervention. He subsequently developed cardiogenic shock and complete heart block. He underwent pacemaker implantation and was considered for advanced heart failure therapy, but unfortunately, he passed away. One patient developed gastrointestinal bleeding and suffered an ischemic stroke during left heart catheterization, so the procedure was aborted. They later passed away due to related complications (Appendices).

**Figure 2:**
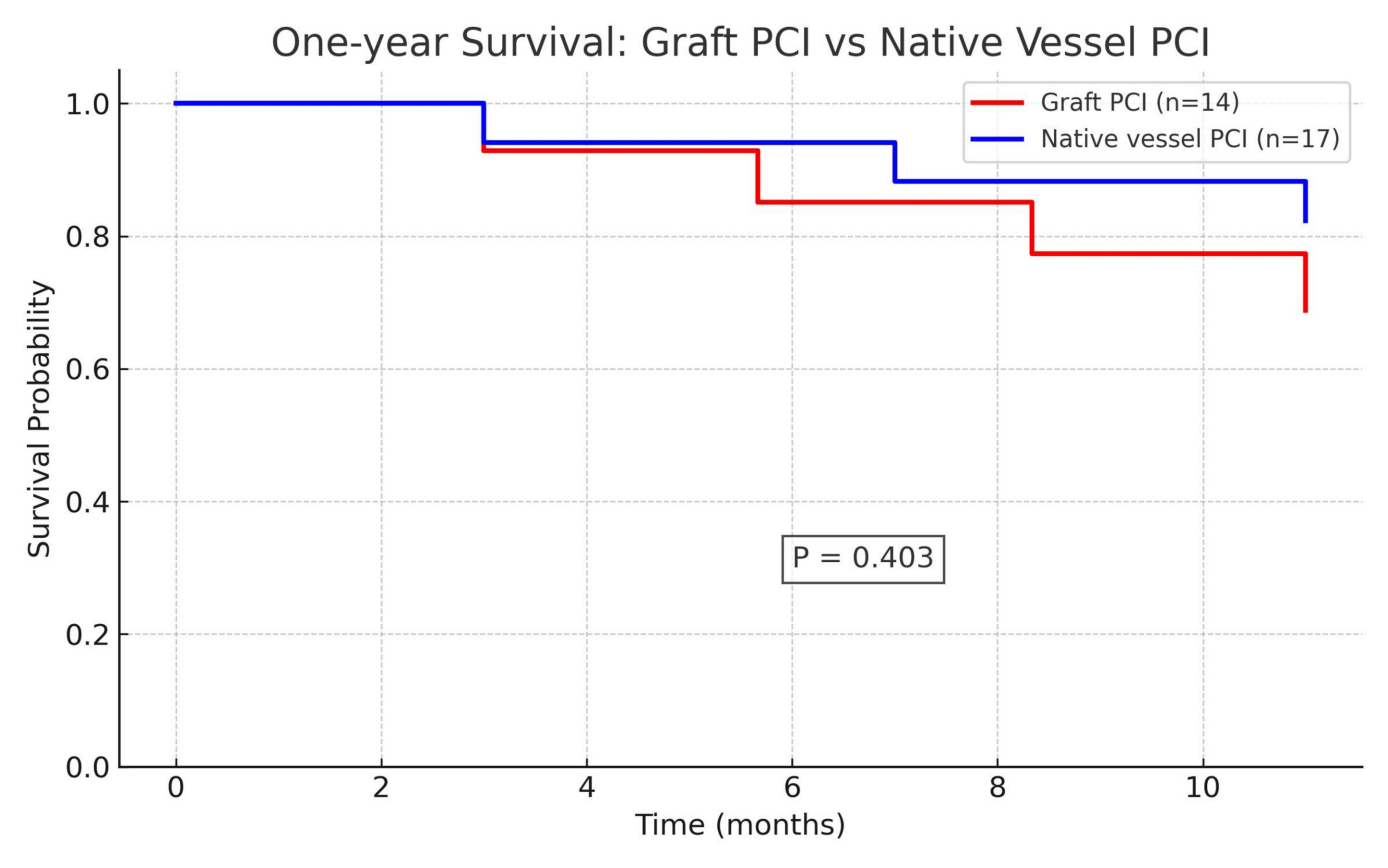
Comparison of one-year survival between graft and native vessel PCI subgroups in the CABG cohort No statistically significant difference between the two groups CABG: coronary artery bypass grafting, PCI: percutaneous coronary intervention

Overall, in-hospital mortality occurred in two (5.7%) cases, with a mean (SD) length of stay (LOS) of 5 (3) days. The one-year survival was 79.1%, with three patients lost to follow-up. When stratified into two groups (native vessel and graft PCI), there were no statistically significant differences in length of stay, post-PCI ejection fraction, MCS requirement, complications during hospitalization, or one-year survival rates (Table 2, Figure 2).

**Table 2:** Outcomes of native vessel versus graft PCI in the CABG cohort a: unpaired t-test, b: Fisher's exact test CABG: coronary artery bypass grafting, LOS: length of stay, PCI: percutaneous coronary intervention, SD: standard deviation

		Native vessel PCI (N=17)	Graft PCI (N=14)	p-value
LOS, days (mean (SD))		5 (4)	3 (2)	0.099^a^
Ejection fraction (mean (SD))		47% (12)	52% (13)	0.28^a^
Use of mechanical circulatory support		2 (11.8%)	2 (14.3%)	1.0^b^
Hospital course complications	Cardiogenic shock	1 (5.9%)	2 (14.3%)	0.59^b^
	Heart failure	2 (11.8%)	1 (7.1%)	1.0^b^
	Acute kidney injury	3 (17.6%)	2 (14.3%)	1.0^b^
	Access site bleeding/hematoma	2 (11.8%)	1 (7.1%)	1.0^b^
	Pneumonia or urinary tract infection	5 (29.4%)	1 (7.1%)	0.37^b^
	Encephalopathy	2 (11.8%)	0 (0%)	0.49^b^
One-year survival		14 (82.4%)	9 (68.8%)	0.40^b^

Comparison with STEMI patients without previous CABG

Compared to patients without CABG, patients with prior CABG were likely to be older and more likely to have hypertension, diabetes mellitus, or hyperlipidemia. The CABG cohort had a greater proportion of African American patients (Table 3).

**Table 3:** Baseline characteristics of patients with STEMI, stratified according to CABG status a: unpaired t-test, b: Fisher's exact test, c: Chi-square test CABG: coronary artery bypass grafting, CKD: chronic kidney disease, EF: ejection fraction, HLD: hyperlipidemia, HTN: hypertension, SD: standard deviation, STEMI: ST-elevation myocardial infarction

	CABG (N=35)	No CABG (N=801)	Test statistic value	p-value
Age (mean±SD) (years)	68±12	60±12	3.86	0.0001^a^
Caucasian	32 (91.43%)	567 (70.78%)	-	0.007^b^
Male	27 (77.14%)	576 (71.91%)	0.46	0.49^c^
HTN	31 (88.57%)	566 (70.66%)	-	0.02^b^
HLD	29 (82.86%)	491 (61.3%)	6.63	0.01^c^
Diabetes	16 (45.71%)	250 (31.21%)	3.25	0.07^c^
CKD	4 (11.43%)	82 (10.23%)	-	0.78^b^
Smoking history	25 (71.43%)	497 (62.05%)	1.26	0.26^c^
EF% (mean±SD)	50±13.6	50.9±13.3	0.17	0.7^a^

Among patients with prior CABG, 31 (88.6%) underwent PCI compared to 651 (81.3%) in the non-CABG group (p=0.35). The most frequent culprit vessel in the non-CABG group was the LAD in 265 (40.7%) patients (Table 4). Thirty-day mortality was 6.25% in the CABG group (with 91.4% follow-up) compared to 5.7% in the non-CABG group (with 90% follow-up) (p=0.9136). MCS was required in six (17.1%) post-CABG patients versus 108 (13.5%) non-CABG patients (p=0.58). The mean length of stay was 5 (3) days in the CABG group compared to 4 (3) days in the non-CABG group (p=0.058).

**Table 4:** Culprit vessel in non-CABG patients with STEMI Expressed as a percentage of those undergoing PCI CABG: coronary artery bypass graft, LAD: left anterior descending artery, LCX: left circumflex artery, LMCA: left main coronary artery, RCA: right coronary artery, STEMI: ST-elevation myocardial infarction

Culprit vessel	Number (%)
LAD	265 (40.7%)
RCA	255 (39.2%)
LCX	60 (9.2%)
Multivessel	43 (6.6%)
Others	24 (3.7%)
LMCA	4 (0.6%)

## Discussion

CABG remains one of the most commonly performed surgical procedures, with approximately 200,000 CABGs performed annually in the United States [9]. With technical advances in both cardiology and cardiac surgery, the long-term survival of patients needing a CABG has increased greatly. However, given that CAD progresses over time, even with optimal medical treatment, many patients with CABG remain at risk for ACS. Thus, understanding the characteristics of STEMI in post-CABG patients is crucial for physicians to provide optimal care.

Overall, patients with prior CABG have been reported to be slightly less than 20% of all patients undergoing PCI [10,11]. Post-CABG patients represent a small proportion of those undergoing PCI for STEMI, with large series reporting between 2.2% and 9.2% of STEMI patients having undergone a prior CABG [1,3,12,13]. We found a similar proportion, with only 4.2% of STEMI patients at our large, regional referral center having prior CABG. This supports prior findings of STEMI being uncommon in patients with CABG; Cader et al. reported STEMI in only 5.4% of a large series of CAG patients undergoing angiography [7]. Several reasons may be hypothesized for this seemingly low incidence of STEMIs in post-CABG patients. Firstly, atherosclerosis of the coronary arteries typically affects the branch points and middle segments, which are bypassed by grafts [14,15]. Secondly, after a CABG, patients received more aggressive management of atherosclerotic risk factors. While this will not completely halt atherosclerosis, it will likely slow it down and reduce STEMI risk [16]. Finally, myocardial ability to adapt to chronic ischemia with microvasculature collateral formation may provide protection against acute transmural infarction even if larger diameter vessels become occluded [17].

We found that 45.2% of post-CABG patients in our cohort required PCI of the bypass grafts. This slightly differs from reports by Welsh et al. [12] and Gharacholou et al. [13], who found that close to half of post-CABG STEMIs involved the graft vessel. It is also a smaller proportion than that reported by Brilakis et al. [10] and Brilakis et al. [11], who reported graft disease in 26.6% and 37.5% of all post-CABG PCIs in two separate database analyses.

Among patients with graft disease, all but one had SVG disease, with the one other patient suffering a LIMA occlusion due to a stent fracture. Again, this is in line with larger series, with Brilakis et al. reporting that an arterial graft was the target for PCI in approximately 2% of post-CABG patients [10]. Atherosclerosis commonly affects SVGs, with more diffuse plaques and thinner, more friable fibrous caps, compared to native arteries [18]. Thus, only about 50%-60% of SVGs last beyond a decade [19]. However, despite these rates of graft failure, probably due to the slow rate of graft occlusion, patients with SVG disease typically present with unstable angina rather than STEMI [7]. On the other hand, LIMA grafts are known for their reliability and do not appear to be affected to the same extent by atherosclerosis, with 10-year patency rates of approximately 90% [20]. LIMA failure, if any, is usually in the early postoperative period, influenced by surgical technique or distal LAD occlusion. Thus, reports of LIMA failure causing STEMI in the long term are few and far between [21,22]. Notably, the LIMA graft was never implicated in our cohort; however, this observation is based on a small sample (one LIMA occlusion due to prior stent fracture) and should not be interpreted as definitive evidence of protection. While arterial grafts such as LIMA are known for long-term patency, the absence of involvement in our series may reflect both the rarity of late LIMA failure and the study’s limited size.

Among the 15 patients in our sample with native vessel disease, six (19.4%) required PCI of multiple vessels, while the RCA was the most common single vessel involved. This differs from the findings of Gharacholou et al., who reported a much smaller proportion of multiple native vessel PCI, as well as the RCA being the most commonly involved artery [13]. Our findings could be attributed to the age and comorbidity burden of our study population, with most of these patients having undergone CABG several years back. We also found that outcomes were similar between the native vessel and graft STEMIs in the CABG group. However, this remains limited by the small size of the subgroups. Prior observational studies have indicated that patients with graft failure have worse peri-procedural and long-term outcomes compared to native vessel STEMI [11]. The results of the PROCTOR trial will likely provide further insight into this interesting subgroup [23].

PCI in post-CABG patients presents unique technical challenges that may contribute to the observed outcomes. These include the complexity of engaging bypass grafts, particularly saphenous vein grafts, which develop diffuse atherosclerosis with friable, complex lesions prone to distal embolization [18]. Additionally, the tortuosity of aged grafts, difficulty in achieving optimal guide catheter support, and challenges in crossing chronic total occlusions in native vessels distal to graft anastomoses contribute to procedural complexity. Furthermore, patients with prior CABG often have extensive coronary disease requiring multivessel intervention, as demonstrated by our finding that 19.3% of native vessel cases required multivessel PCI.

When compared with patients without prior CABG, we found that post-CABG patients were older and had greater comorbidity burden, although these comparisons were unadjusted. This is unsurprising and in line with prior comparisons [10-12]. There was a smaller proportion of African Americans, which could be reflective of prior and/or persisting racial disparities among patients who have received CABG [24,25]. On the other hand, it may also represent that African American patients have better outcomes after CABG with less likelihood of STEMI presentation. Finally, patients with prior CABG, in our unadjusted analysis, had longer LOS compared to patients without prior CABG. This aligns with prior reports [1,5,6,12], which have shown that post-CABG patients undergoing PCI have worse outcomes compared to no-CABG patients, particularly in SVG PCIs [6,10-12,26]. Potential reasons for this include the typically greater comorbidity burden in post-CABG patients and the technical challenges of PCIs in post-CABG STEMIs [12]. While we did not assess long-term outcomes, in matched analyses, long-term survival of post-CABG patients has not been demonstrably worse than non-CABG patients after PCI for STEMIs, although they have been reported to suffer greater major adverse cardiovascular events (MACE) [12]. Our study provides further valuable data on the clinical and angiographic characteristics of STEMI in patients with prior CABG.

Limitations and future directions

Our study has several important limitations. First, we included patients presenting to a single large academic center, which may limit generalizability, given that only one region was involved, with more medically complex patients. Second, the small post-CABG cohort (n=35), due to its relative rarity, limited statistical power and precluded multivariable analysis. Our comparisons were unadjusted, therefore subject to potential confounding. Third, the transition from conventional to high-sensitivity troponin during the study period may have affected diagnostic consistency. Finally, the retrospective nature of angiographic review, while confirmed by chart documentation, may be subject to interpretation bias, particularly for complex lesion morphology and TIMI flow assessments.

Our findings highlight the need for multicentric studies with appropriate adjustment for confounders, along with longer-term studies. Future studies should utilize multivariable adjustment or matched cohort designs to better control for confounding variables and identify independent associations. Our study did not assess medication use differences, such as statin intensity or dual antiplatelet therapy adherence, between CABG and non-CABG cohorts, which may also influence outcomes. This remains a limitation given their known impact on cardiovascular risk and should be explored in future investigations.

Future studies should focus on elucidating the underlying mechanisms for post-CABG STEMI, through advanced imaging modalities and biomarker studies, along with the potential protective effects of CABG on further STEMI. Understanding whether collateral circulation development, altered plaque morphology, or aggressive secondary prevention strategies account for the reduced STEMI incidence could inform clinical decision-making and risk stratification algorithms. Our study is one of the few single-center studies to report on the angiographic and clinical features of STEMI in post-CABG patients over a prolonged period. The real-world insights from this diverse, longitudinal cohort contribute meaningfully to a limited evidence base.

## Conclusions

Our findings suggest that STEMI is uncommon after CABG. Post-CABG patients were typically older, raising the possibility of a protective effect of surgical revascularization against plaque rupture. STEMI in these patients occurred almost equally in SVG and native vessels, with similar outcomes observed. While we provide valuable data on this uncommon clinical scenario, larger, multicentric studies with appropriate risk adjustment remain needed to elucidate long-term outcomes.

## Appendices

The clinical characteristics of all patients with CABG-STEMI are presented in Table 5.

**Table 5:** Clinical characteristics of all patients with CABG-STEMI a: unable to cross the lesion, b: patient passed away, c: patient had a stroke, d: IABP placed, no PCI required: LIMA was atretic, but not culpable CABG: coronary artery bypass graft, D1: first diagonal artery, EKG: electrocardiography, IABP: intra-aortic balloon pump, LBBB: left bundle branch block, LCX: left circumflex artery, LIMA: left internal mammary artery, LAD: left anterior descending artery, OM: obtuse marginal artery, PCI: percutaneous coronary intervention, PDA: posterior descending artery, RBBB: right bundle branch block, RCA: right coronary artery, RIMA: right internal mammary artery, STEMI: ST-elevation myocardial infarction, SVG: saphenous vein grafts, TIMI: thrombolysis in myocardial infarction, u/n: unavailable

Serial number	Age	Duration since CABG (years)	Chest pain	Diaphoresis	Cardiac arrest	EKG changes/wall affected	Culprit vessel	Patent grafts	Chronic occlusion	Mechanical support	Post-revascularization TIMI score
1	68	1 day	Yes	No	No	Inferior, lateral walls	D1	LIMA-LAD, SVG-OM1	No	No	3
2	56	23	Yes	Yes	No	Anterior wall	RCA	LIMA-LAD	No	No	3
3	60	8	Yes	No	No	LBBB, anterior wall	SVG-D1	SVG-LAD, SVG-OM1, SVG-OM2	No	Yes	3
4	55	0.5	Yes	No	No	Inferior wall	SVG-PDA	LIMA-LAD, SVG-D1 SVG-OM1	No	No	3
5	51	11	Yes	Yes	No	Inferior, posterior, apical walls	No-PCI^a^	LIMA-LAD	SVG-RCA SVG-OM1	No	0
6	50	11	Yes	No	No	Anterolateral wall	LIMA-LAD anastomosis	SVG-OM-1	SVG-D1	No	3
7	85	25	Yes	Yes	No	Inferior wall	RCA	LIMA-LAD, SVG-OM1	No	No	3
8	79	13	Yes	No	No	RBBB, inverted T-waves	SVG-PDA	LIMA-LAD, SVG-D1	No	No	3
9	72	23	Yes	No	No	Anterior wall	Proximal LAD	LIMA-LAD	SVG-RCA	No	3
10	91	24	Yes	No	No	Inferior wall	RCA	SVG-LAD	No	No	3
11	72	1	No	No	Yes	Anterior wall	LCX, OM1	LIMA-LAD, SVG-D1	No	No	3
12	51	3	No	No	Yes	Inferior wall	No PCI^b^	LIMA-LAD	SVG-D1, SVG-OM1 SVG-PDA	Yes	u/n
13	83	9	No	No	Yes	Anterior wall	LM, LCX, OM2	LIMA-LAD, SVG-RCA SVG-D1	SVG-OM1	No	3
14	50	11	Yes	Yes	No	Anterolateral wall	SVG-OM1	LIMA-LAD, SVG-PDA	No	No	2
15	81	24	Yes	No	No	Inferior wall	SVG-PDA	LIMA-LAD, SVG-D1 SVG-OM1	No	No	3
16	67	1	Yes	No	No	Anterior wall	Proximal LAD	LIMA-LAD	No	Yes	3
17	68	u/n	Yes	No	No	Inferior wall	Distal PDA, OM	LIMA-LAD, RIMA-PDA	No	No	3
18	71	9	Yes	No	No	Inferior wall	RCA	LIMA-LAD, SVG-OM1	SVG-OM2	No	3
19	83	25	Yes	No	No	Inferior wall	LCX	LIMA-LAD	SVG-RCA	No	3
20	79	25	No	No	Yes	Inferior septal, posterior walls	Proximal LAD	LIMA-LAD	SVG-Ramus SVG-OM1	No	3
21	84	13	Yes	No	No	Posterior wall	SVG-OM1	LIMA-LAD	No	No	3
22	67	6	Yes	No	No	Inferior wall	LCX	LIMA-LAD	SVG-RCA SVG-OM1	No	3
23	59	u/n	Yes	No	No	Inferior wall	No PCI^c^	LIMA-LAD SVG-OM1	No	No	u/n
24	68	12	Yes	No	No	Anterior wall	Proximal LAD	RIMA-LAD	LIMA-LCX	No	3
25	66	19	Yes	No	No	Inferior wall	LCX, OM2	SVG-PDA	LIMA-LAD	No	3
26	50	7	Yes	No	No	Anterolateral wall	SVG-RCA	LIMA-LAD, SVG-OM1	No	No	3
27	56	9	Yes	Yes	No	Posterior wall	SVG-OM1	LIMA-LAD	No	No	3
28	63	15	Yes	No	No	Inferior wall	SVG-RCA	SVG-LAD	No	No	3
29	64	u/n	No	No	No	Inferior wall	No PCI^d^	LIMA-LAD, SVG-OM1	No	Yes	u/n
30	57	6	Yes	No	No	Inferior wall	RCA	LIMA-LAD, SVG-RCA SVG-D1, SVG-OM1	No	No	3
31	82	20	Yes	Yes	No	Inferior wall	SVG-RCA	LIMA-LAD	No	No	1
32	70	15	Yes	No	No	Inferior wall	SVG-RCA	LIMA-LAD, SVG-OM1	No	No	3
33	69	3	Yes	No	No	Inferior wall	RCA	LIMA-LAD	SVG-OM1	No	3
34	74	11	Yes	Yes	No	Inferior wall	SVG-RCA, SVG-D1	LIMA-LAD	SVG-OM1	Yes	3
35	88	12	Yes	Yes	No	Inferior wall	SVG-Ramus	LIMA-LAD	No	No	3
